# Clinical Characteristics of Ratborne Seoul Hantavirus Disease

**DOI:** 10.3201/eid2502.181643

**Published:** 2019-02

**Authors:** Jan Clement, James W. LeDuc, Lorraine M. McElhinney, Jean-Marc Reynes, Marc Van Ranst, Charles H. Calisher

**Affiliations:** University of Leuven, Leuven, Belgium (J. Clement, M. Van Ranst);; University of Texas Medical Branch, Galveston, Texas, USA (J.W. LeDuc);; Animal and Plant Health Agency, Weybridge, UK (L.M. McElhinney);; Institut Pasteur, Paris, France (J.-M. Reynes);; Colorado State University, Fort Collins, Colorado, USA (C.H. Calisher)

**Keywords:** Seoul orthohantavirus, SEOV, viruses, signs, symptoms, acute kidney injury, proteinuria, microhematuria, ratborne Seoul hantavirus disease, hemorrhagic fever with renal syndrome, HFRS, hantavirus cardiopulmonary syndrome, HCPS, diagnosis, rats, zoonoses

## Abstract

Although Seoul orthohantavirus is the only globally spread hantavirus pathogen, few confirmed human infections with this virus have been reported in Western countries, suggesting lower medical awareness of the milder, transient, and often chameleon-like symptoms of this zoonosis. We describe lesser known clinical and laboratory characteristics to help improve underreporting of this virus.

Recent reports from several Western countries, including the United States, have described an ill-known hantavirus disease, commonly called hemorrhagic fever with renal syndrome (HFRS), induced by Seoul orthohantavirus (SEOV) and spread by infected wild, laboratory, and pet rats. These reports might bring to an end the long-maintained concept that human hantavirus infections were to be distinguished between HFRS in the Old World and hantavirus cardiopulmonary syndrome (HCPS) in the New World (*1*). New World human hantavirus illnesses already described in 1993 were HFRS cases, not HCPS cases, in leptospirosis-suspected patients with acute kidney injury (AKI) and thrombocytopenia, whereas the earliest characterized hantavirus pathogen in the United States was not Sin Nombre orthohantavirus (SNV) but SEOV, isolated from a wharf rat in Philadelphia in 1984 (*1*).

Geographic distributions of most hantaviruses are limited to the biotope area of their respective natural hosts. The exception is SEOV, which is distributed worldwide, because of the omnipresence of its synanthropic hosts, the brown (*Rattus norvegicus*) and black (*R. rattus*) rats. SEOV likely arose in northern China, then spread to Europe and subsequently to the Americas in the 18th century (*2**,**3*). By the early 1980s, SEOV-infected rats were detected in Asia, Africa, Europe, and the Americas (*4*). SEOV strains are all closely related, probably reflecting the recent worldwide spread of rats, speculated to be driven by introduction via sea ports and railways, and resulting now in chronic rat SEOV endemicity (*2**,**3*).

Human SEOV infections have long been recognized in all those areas, including the United States (*1**,**4*). Consequently, it is counterintuitive to expect that the scant number of SEOV-induced HFRS cases reported so far in Western countries reflects the actual situation, in stark contrast to the many SEOV cases documented in Asia, particularly in South Korea and China, both registering >90% of all hantavirus cases worldwide, of which >25% are caused by SEOV (*3**,**5*). We stress the diagnostic challenge inherent to milder (case-fatality rate ± 1%), transient, and atypical hantavirus infections, some of which might represent SEOV infections.

HFRS and SEOV HFRS are characterized by the same prodromal symptoms for 3–5 days as for HCPS: fever, myalgia, malaise, and gastrointestinal discomfort (*5*). All HFRS forms show not only AKI, ranging from strictly normal to severely impeded renal function, but also rapidly changing degrees of proteinuria, microhematuria, and thrombocytopenia (*5*). However, established presence or absence of initial proteinuria and microhematuria has not been investigated so far in large-scale SNV HCPS studies.

Proteinuria and microhematuria, although transient, are considered severity indicators for HFRS (*5**,**6*). The rapidity of increasing/decreasing proteinuria is virtually pathognomonic for HFRS and was noted as early as 1964. Epidemics of a then ill-defined fever called epidemic hemorrhagic fever, which was later proven serologically to be a wild rat–induced HFRS (*7*), was present principally in the back alleys of Osaka, Japan, and characterized by marked but transient proteinuria that peaked in 32 case-patients on day 6 postonset of symptoms and disappeared completely on day 7 in mild cases and on approximately day 12 in those with severe AKI (*8*). Moreover, severity of proteinuria was found to be predictive of overall epidemic hemorrhagic fever clinical severity, as confirmed 53 years later in 70 case-patients infected with Puumala orthohantavirus; proteinuria (30% of nephrotic range), which peaked on day 5 postonset of symptoms, decreased almost completely on day 11, whereas serum creatinine levels peaked on day 9 (*6*).

Until recently, sudden AKI with nephrotic-range proteinuria and microhematuria was considered a rare nephrologic triad in previously healthy young adults; these adults constituted most HFRS case-patients. AKI with proteinuria, after acute tubular necrosis, is sometimes ascribed to the effect of nonsteroidal antiinflammatory drugs (NSAIDs). Because NSAIDs are often prescribed for the influenza-like myalgiae preceding HFRS, these drugs can obscure the real ensuing cause of AKI (*5*). However, NSAIDs do not induce thrombocytopenia or the other biochemical hallmarks of HFRS (Appendix Table).

In contrast to severe HCPS caused by SNV or Andes orthohantavirus (ANDV), HFRS cases can be a diagnostic puzzle involving several swollen organs and including the lungs (*5*). The 2 earliest documented hantavirus infections in Peru were 2 SEOV HFRS case-patients confirmed by reverse transcription PCR, but both case-patients had fatal HFRS with HCPS (*9*), thus further blurring the boundaries between the 2 syndromes. In Southeast Asia, where the wild rat was and is the major reservoir for pathogenic hantaviruses, HFRS with liver involvement, imitating virus hepatitis, was moreover proposed as a new clinical entity (*10*).

Finally, laboratory confirmation of diagnosis, even by an expert clinician, can be confounded by use of current commercial serologic assays that use antigens having weak or no cross-reactivity with murine SEOVs, such as European arvicoline Puumala orthohantavirus or American sigmodontine SNV/Andes orthohantavirus. Close attention should be paid to the multifaceted diagnosis of SEOV infection (Appendix Table) in patients exposed to brown or black rat excreta, including pet rat owners.

Appendix Additional information on clinical characteristics of ratborne Seoul hantavirus disease.
