# Supplementary material for: Clinical Characteristics of Ratborne Seoul Hantavirus Disease
Source: Emerg Infect Dis. 2019 Feb;25(2):387–8. doi: 10.3201/eid2502.181643 (PMC6346471; doi:10.3201/eid2502.181643)
Supplement: Appendix — Additional information on clinical characteristics of ratborne Seoul hantavirus disease. [file 18-1643-Techapp-s1.pdf]

Article DOI: https://doi.org/10.3201/eid2502.181643

Clinical Characteristics of Ratborne Seoul Hantavirus Disease

Appendix

Appendix Table. Clinical characteristics of ratborne Seoul hantavirus disease*

**Clinical symptoms common for HFRS, but also for HCPS**
Premonitory complaints (3- 5 d): sudden high fever, malaise, vomiting and diarrhea, severe **gastrointestinal pains**, mimicking acute appendicitis (*1–4*)
Headache and **influenza-like myalgiae**, but no premonitory upper airways symptoms evoking influenza: no rhinorrhea or throat ache
Initial eye pain and periorbital edema
Facial flushing, pharyngeal congestion (*5*)
(Micro-)hemorrhages, sometimes limited to conjunctival suffusion or intraoral petechiae† (*5–8)*
Dry cough, followed by **dyspnea** (*7–14)*, outspoken and rapidly worsening in HCPS
Short-lived pseudo nephrotic syndrome:† despite massive proteinuria and frank hypoalbuminemia, conspicuous absence of generalized pitting edema (*4,12*), hyperlipidemia, and thrombotic tendency
**Clinical symptoms reported for HFRS, not for HCPS**
**Acute myopia** (2–3 d) as a virtually pathognomonic, but transient sign (*6,7,14*); rarely acute bilateral glaucoma or retinal hemorrhage (*15*)
Severe **flank pain** (lumbalgia), sometimes unilateral, mimicking a renal colic (*16,17*)
Rarely acute bilateral glaucoma or retinal hemorrhage (*15*)
Transient acalculous acute cholecystitis provoking right upper quadrant pain and a positive Murphy sign (*18–20)*; considered a general severity sign (*21*)
Paradoxical sinusal bradycardia (<90 bpm), despite fever >38°C (*6,7*)
**Laboratory anomalies mainly reported for HFRS**
Initial **thrombocytopenia** is the earliest and most constant sign‡ (*4–6*)
**Urine spot PCR§ >0.11** plus microhematuria; early (mostly before hospitalization) and **rapidly evolving**, but cardinal sign, easy, and cheap to assess day-by-day
Hyponatremia and hypoalbuminemia, predicting clinical severity‡ (*5,8,12,22*)
Highly increased levels of LDH, and particularly of CRP and PCT, mimicking hemolysis (*18,22*) or a bacteria, rather than a virus, infection‡ (*6–8*)
Lipid paradox: low acute cholesterolemia (particularly decreased high-density lipoprotein–cholesterol levels), contrasting with fasting hypertriglyceridemia, both transitory (*7,18,23,24)*
Serum creatinine levels might remain initially (*18,20*) or constantly (*4,7,25–27*) at **standard levels**, or barely and briefly increased (*28*)
Slight-to-frank hypokalemia, despite often clearly impeded renal function (*8,29*)
**Ultrasound anomalies, reported more for HFRS than for HCPS**
**Third-space, protein-rich fluid effusions‡** (pleuritis, pericarditis, ascites)
**Longitudinal renal diameter >11 cm¶** (*17,18*), swollen cortex with echodensity greater than or equal to that for liver. Rare but virtually present or or absent pathognomonic sign: perirenal fluid rim
Transient hepatomegaly and splenomegaly (*4,8,17*)
AAC with mostly distended large gallbladder, and thickened (>4.5 mm) edematous gallbladder wall (*18–21*)
**Liver involvement suggesting Seoul virus (SEOV) involvement**
Aminotransferase levels increased to 10–20 times (or more) above the standard level (*4,12,26)* versus mild or no transaminitis in other HFRS forms (*6–8*); concomitant renal function impediment might be conspicuously absent (*4,7)*
Rarely: icterus; can be pronounced, but is rapidly self-remitting (*7,30*)

*All signs or symptoms are in present or absent chronologic order of appearance. Bold indicates a high diagnostic value. A general common feature is rapid self-remittance within days or weeks without leaving any sequelae (*6*). Ultrasound-documented renal shrinking within days is highly suggestive for HFRS. AAC acute cholecystitis; bpm, beats per minute; CRP, C-reactive protein; HCPS, hantavirus cardiopulmonary syndrome; HFRS, hemorrhagic fever with renal syndrome; LDH, lactate dehydrogenase; PCR, protein-to-creatinine ratio; PCT, procalcitonin.
†Rare to absent in HCPS, except in Andes virus (ANDV)–induced forms (*5*).
‡Also present in HCPS.
§Urine spot PCR, or urinary protein-to-creatinine ratio, is calculated in milligrams per deciliter or grams per liter; standard value for adults is <0.11. Urine spot PCR is also a surrogate for 24-h protein excretion in g/day, indicating that a urinary protein-to-creatinine ratio >3.5 is nephrotic-range proteinuria or equivalent to a dipstick value of +++(+).
¶Standard range 11–12 cm depending on body size. Ultrasound-documented renal shrinking within days is highly suggestive for HFRS

References

1. Zetterholm SG. Nephrititis simulating acute abdomen [in Swedish]. Lakartidningen. 1934;31:425–6.

2. Myhrman G. A renal disease with particular symptoms [in Swedish].7 Medicinsk Tidskrift. 1934;7:793–4.

3. Latus J, Fritzenkötter M, Schmidt-Chanasit J, Tenner-Racz K, Leibold T, Kimmel M, et al. Hantavirus and acute appendicitis—the diagnosis behind the diagnosis? J Clin Virol. 2012;53:156–8. PubMed http://dx.doi.org/10.1016/j.jcv.2011.11.003

4. Swanink C, Reimerink J, Gisolf J, de Vries A, Claassen M, Martens L, et al. Autochthonous human case of Seoul virus infection, the Netherlands. Emerg Infect Dis. 2018;24:2158–63. PubMed http://dx.doi.org/10.3201/eid2412.180229

5. Peters CJ, Khan AS. Hantavirus pulmonary syndrome: the new American hemorrhagic fever. Clin Infect Dis. 2002;34:1224–31. PubMed http://dx.doi.org/10.1086/339864

6. Pal E, Korva M, Resman Rus K, Kejžar N, Bogovič P, Kurent A, et al. Sequential assessment of clinical and laboratory parameters in patients with hemorrhagic fever with renal syndrome. PLoS One. 2018;13:e0197661. PubMed http://dx.doi.org/10.1371/journal.pone.0197661

7. Colson P, Damoiseaux P, Brisbois J, Duvivier E, Levecque P, Roger JM, et al. Epidemic of hantavirus disease in Entre-Sambre-et-Meuse: year 1992–1993. Clinical and biological aspects [in French]. Acta Clin Belg. 1995;50:197–206. PubMed http://dx.doi.org/10.1080/17843286.1995.11718447

8. Dreshaj S, Ajazaj L, Hasani N, Halili B, Ponosheci A, Jakupi X. A nonfatal case of Dobrava hantavirus hemorrhagic fever with renal syndrome combined with hantavirus cardiopulmonary syndrome. J Glob Infect Dis. 2018;10:22–5. PubMed http://dx.doi.org/10.4103/jgid.jgid_12_17

9. Vollmar P, Lubnow M, Simon M, Müller T, Bergler T, Alois P, et al. Hantavirus cardiopulmonary syndrome due to Puumala virus in Germany. J Clin Virol. 2016;84:42–7. PubMed http://dx.doi.org/10.1016/j.jcv.2016.10.004

10. Stuart LM, Rice PS, Lloyd G, Beale RJ. A soldier in respiratory distress. Lancet. 1996;347:30. PubMed http://dx.doi.org/10.1016/S0140-6736(96)91560-3

11. Schütt M, Meisel H, Krüger DH, Ulrich R, Dalhoff K, Dodt C. Life-threatening Dobrava hantavirus infection with unusually extended pulmonary involvement. Clin Nephrol. 2004;62:54–7. PubMed http://dx.doi.org/10.5414/CNP62054

12. Roig IL, Musher DM, Tweardy DJ. Severe pulmonary involvement in a case attributed to domestically acquired Seoul hantavirus in the United States. Clin Infect Dis. 2012;54:91–4. PubMed http://dx.doi.org/10.1093/cid/cir748

13. Taori SK, Jameson LJ, Campbell A, Drew PJ, McCarthy ND, Hart J, et al. UK hantavirus, renal failure, and pet rats. Lancet. 2013;381:1070. PubMed http://dx.doi.org/10.1016/S0140-6736(13)60599-1

14. Hautala N, Kauma H, Vapalahti O, Mähönen SM, Vainio O, Vaheri A, et al. Prospective study on ocular findings in acute Puumala hantavirus infection in hospitalised patients. Br J Ophthalmol. 2011;95:559–62. PubMed http://dx.doi.org/10.1136/bjo.2010.185413

15. Zimmermann A, Lorenz B, Schmidt W. Bilateral acute angle-closure glaucoma due to an infection with hantavirus [in German]. Ophthalmologe. 2011;108:753–8. PubMed http://dx.doi.org/10.1007/s00347-010-2311-8

16. Van Ypersele de Strihou C. Nephrology forum: acute oliguric intersititial nephritis. Kidney Int. 1979;16:751–65. PubMed http://dx.doi.org/10.1038/ki.1979.192

17. van Ypersele de Strihou C, van der Groen G, Desmyter J. Hantavirus nephropathy in western Europe: ubiquity of hemorrhagic fevers with renal syndrome. Adv Nephrol Necker Hosp. 1986;15:143–72. PubMed

18. Keyaerts E, Ghijsels E, Lemey P, Maes P, Zachée P, Daelemans R, et al. Plasma-exchange-associated IgM-negative hantavirus disease after a camping holiday in southern France. Clin Infect Dis. 2004;38:1350–6. PubMed http://dx.doi.org/10.1086/383311

19. Fröhlich R, Römmele U. Acalculous cholecystitis in hantavirus infections [in German]. Dtsch Med Wochenschr. 2013;138:1255–8. PubMed

20. Mahmud M, Winkelmann C, Harendza S. Fulminant acute kidney injury after cholecystectomy in a 45-year-old female patient [in German]. Internist (Berl). 2018. PubMed

21. Kim YO, Chun KA, Choi JY, Yoon SA, Yang CW, Kim KT, et al. Sonographic evaluation of gallbladder-wall thickening in hemorrhagic fever with renal syndrome: prediction of disease severity. J Clin Ultrasound. 2001;29:286–9. PubMed http://dx.doi.org/10.1002/jcu.1035

22. Clement J, Lee APK, Verpooten GA, Laenen L, Vergote V, De Samblanx H, et al. Acute hantavirus infection presenting as haemolytic-uraemic syndrome (HUS): the importance of early clinical diagnosis. Eur J Clin Microbiol Infect Dis. 2018;37:135–40. PubMed http://dx.doi.org/10.1007/s10096-017-3113-6

23. Clement J, Colson P, Saegeman V, Lagrou K, Van Ranst M. 'Bedside assessment' of acute hantavirus infections and their possible classification into the spectrum of haemophagocytic syndromes. Eur J Clin Microbiol Infect Dis. 2016;35:1101–6. PubMed http://dx.doi.org/10.1007/s10096-016-2638-4

24. Maes P, Clement J, Groeneveld P, Colson P, Huizinga T, Van Ranst M. TNF-alpha genetic predisposing factors can influence clinical severity in nephropathia epidemica. Viral Immunol. 2006;19:558–64. PubMed http://dx.doi.org/10.1089/vim.2006.19.558

25. Alexeyev OA, Baranov BA. Puumala virus infection without signs of renal involvement. Scand J Infect Dis. 1993;25:525–7. PubMed http://dx.doi.org/10.3109/00365549309008537

26. Reynes J-M, Carli D, Bour J-B, Boudjeltia S, Dewilde A, Gerbier G, et al. Seoul virus infection in humans, France, 2014–2016. Emerg Infect Dis. 2017;23:973–7. PubMed http://dx.doi.org/10.3201/eid2306.160927

27. Kerins JL, Koske SE, Kazmierczak J, Austin C, Gowdy K, Dibernardo A, et al.; Seoul Virus Working Group; Canadian Seoul Virus Investigation Group (Federal); Canadian Seoul Virus Investigation Group (Provincial); Contributors. Outbreak of Seoul vrus among rats and rat owners—United States and Canada, 2017. MMWR Morb Mortal Wkly Rep. 2018;67:131–4. PubMed http://dx.doi.org/10.15585/mmwr.mm6704a5

28. Fill MA, Mullins H, May AS, Henderson H, Brown SM, Chiang CF, et al. Notes from the field: multiple cases of Seoul virus infection in a household with infected pet rats—Tennessee, December 2016–April 2017. MMWR Morb Mortal Wkly Rep. 2017;66:1081–2. PubMed http://dx.doi.org/10.15585/mmwr.mm6640a4

29. Clement J, Maes P, Van Ranst M. Acute kidney injury in emerging, non-tropical infections. Acta Clin Belg. 2007;62:387–95. PubMed http://dx.doi.org/10.1179/acb.2007.058

30. Monteiro J, Mesquita M, Alves M, Filipe AR. Hemorrhagic fever with renal syndrome: first clinical case diagnosed in Portugal [in Portuguese]. Separata da Revista Portuguesa de Doenas Infecciosas. 1993;16:209–14.
